# Current Strategies to Combat Cisplatin-Induced Ototoxicity

**DOI:** 10.3389/fphar.2020.00999

**Published:** 2020-07-03

**Authors:** Dehong Yu, Jiayi Gu, Yuming Chen, Wen Kang, Xueling Wang, Hao Wu

**Affiliations:** ^1^ Department of Otolaryngology-Head and Neck Surgery, Shanghai Ninth People’s Hospital, School of Medicine, Shanghai Jiao Tong University, Shanghai, China; ^2^ Ear Institute, School of Medicine, Shanghai Jiao Tong University, Shanghai, China; ^3^ Shanghai Key Laboratory of Translational Medicine on Ear and Nose Diseases (14DZ2260300), Shanghai, China

**Keywords:** cisplatin-induced ototoxicity, drug delivery systems, animal models, clinical trials

## Abstract

Cisplatin is widely used for the treatment of a number of solid malignant tumors. However, ototoxicity induced by cisplatin is an obstacle to effective treatment of tumors. The basis for this toxicity has not been fully elucidated. It is generally accepted that hearing loss is due to excessive production of reactive oxygen species by cells of the cochlea. In addition, recent data suggest that inflammation may trigger inner ear cell death through endoplasmic reticulum stress, autophagy, and necroptosis, which induce apoptosis. Strategies have been extensively explored by which to prevent, alleviate, and treat cisplatin-induced ototoxicity, which minimize interference with antitumor activity. Of these strategies, none have been approved by the Federal Drug Administration, although several preclinical studies have been promising. This review highlights recent strategies that reduce cisplatin-induced ototoxicity. The focus of this review is to identify candidate agents as novel molecular targets, drug administration routes, delivery systems, and dosage schedules. Animal models of cisplatin ototoxicity are described that have been used to evaluate drug efficacy and side effect prevention. Finally, clinical reports of otoprotection in patients treated with cisplatin are highlighted. For the future, high-quality studies are required to provide reliable data regarding the safety and effectiveness of pharmacological interventions that reduce cisplatin-induced ototoxicity.

## Highlights

Mechanisms that may underlie cisplatin-induced ototoxicity include excessive ROS, inflammation, cellular uptake of cisplatin, and autophagy, which result in cell death.

Preclinical results have demonstrated numerous interventions that reduce cisplatin-induced ototoxicity. These include small molecular compounds and various drug delivery systems. Potential candidates are nanoparticles and hydrogels that can be delivered via different administration routes and by different dose schedules.

Although 2 recent phase III clinical trials (Freyer et al., 2017; Brock et al., 2018) have shown reduced incidence and severity of cisplatin-related ototoxicity, further clinical trials are required.

## Introduction

Cisplatin is a widely used chemotherapeutic agent with a high degree of ototoxicity and an average incidence of over 60% (Karasawa and Steyger, 2015). Ototoxicity depends on several factors, including patient age, cumulative dose of cisplatin, and genetic susceptibility (Landier, 2016; Lanvers-Kaminsky and Ciarimboli, 2017). The effect of ototoxicity is greatest for children due to potential delays in education and psychosocial development (Knight et al., 2005).

Ototoxicity encompasses the cochlea (cochleotoxicity) and the vestibule (vestibulotoxicity). Cochleotoxicity manifests as hearing loss and tinnitus with substantial impact on communication (Watts, 2019). Hearing loss is bilateral, progressive, and irreversible, with impairment beginning at higher frequencies and extending to lower ones (Rybak, 2007).

The underlying basis for ototoxicity can be multifactorial. Cellular damage may be reflected by changes in functional measurements such as endocochlear potential (EP), auditory brainstem response (ABR), and distortion product otoacoustic emissions (DPOAE) (Gentilin et al., 2019). The underlying mechanistic basis for ototoxicity, which could serve as a starting point for rational drug design, is unknown. Extensive systemic and local therapies to prevent hearing loss have been evaluated. However, none have been approved by the FDA.

This review summarizes the generally accepted underlying basis for cisplatin-induced ototoxicity, explores experimental animal strategies to limit ototoxicity, and introduces limited clinical trials that evaluate approaches to reduce ototoxicity.

## Mechanisms of Cisplatin-Induced Ototoxicity

he mechanistic basis for ototoxicity induced by cisplatin is not fully understood. However, cisplatin is known to damage the organ of Corti, the spiral ganglion, and the stria vascularis as primary targets. Experimental studies have shown that cellular uptake of cisplatin, oxidative stress, inflammation, apoptosis, and autophagy can play major roles in the pathogenesis of cisplatin-induced ototoxicity.

### Cisplatin Uptake

Recent evidence suggests that long-term retention of cisplatin in the inner ear drives cisplatin ototoxicity (Breglio et al., 2017). It has been reported that systemic gentamicin is trafficked via the endolymph into cochlear cells (Wang and Steyger, 2009; Li and Steyger, 2011). If cisplatin acts in a similar manner, two pathways are involved; passive diffusion, and active uptake by a number of membrane-embedded proteins (Hall et al., 2008). These include mechanoelectrical transducer (MET) channels (Brock et al., 2012), copper transporters (Ctr1, Ctr2), organic cation transporter (OCT2), and transient receptor potential channel (TRPV1), which may contribute to cisplatin influx (Waissbluth and Daniel, 2013). However, there is increasing skepticism regarding protein-mediated cisplatin uptake for the following reasons; (1) the irreversible binding of platinum to CTR1 protein (Ivy and Kaplan, 2013), (2) the unsaturation of platinum uptake *via* CTR1 (Hall et al., 2008), and (3) the absence of OCT2 in the stria vascularis which denies uptake of cisplatin by vascular networks (Hellberg et al., 2015). Rather, the neutrally charged cisplatin may passively diffuse across the lipid phase of the cell membrane and into the cytoplasm where chloride ligands are replaced through an aquation reaction. The reaction would convert cisplatin to a positively charged diaquo derivative with significantly reduced permeability (Eljack et al., 2014). As described below, the aquated form could then bind to and damage a variety of macromolecules including RNA, proteins, membrane phospholipids, and DNA.

### Imbalance of Antioxidant Defense System

Oxygen is essential to energy generation by a series of one-electron reductions in the mitochondrial electron transport chain. Superoxide radicals (O_2_^-^), hydrogen peroxide (H_2_O_2_), and hydroxyl radicals (OH^-^) are generated and known as reactive oxygen species (ROS) (Song et al., 2011). ROS generation may contribute to hearing loss due to cisplatin. NADPH oxidase (NOX)3, which is at least 50-fold higher in the inner ear than in any other tissue (e.g., fetal kidney, brain, skull) is strongly induced by cisplatin (Banfi et al., 2004; Karasawa and Steyger, 2015). Knockdown of this gene by siRNA administration to the middle ear ameliorates hearing loss (Mukherjea et al., 2010; Rybak et al., 2012). Another source of ROS in the cochlea is xanthine oxidase (XO) (Lynch et al., 2005). A combination of XO and its substrate hypoxanthine (HX) induce a rise in cytosolic free calcium, resulting in a change in outer hair cell (OHC) motility (Ikeda et al., 1993). Moreover, increased ROS opens Ca^2+^-permeable channels in sarcoplasmic/endoplasmic reticulum (SR/ER) membranes (IP_3_R and RyR) and plasma membranes (TRPV1, L-type, and T-type calcium channels) (Song et al., 2011), leading to an increase in cytosolic calcium levels and eventual apoptotic and autophagic cell death (Pinton et al., 2008).

Enhanced cellular ROS production is balanced by oxygen radical scavengers and antioxidant enzymes that neutralize oxidative damage and include superoxide dismutase (SOD), catalase (CAT), glutathione peroxidase (GSH.Px), and glutathione (GSH) (Sheth et al., 2017). Depletion of antioxidants after exposure to cisplatin has been found in most studies (Lautermann et al., 1997; Rybak et al., 2000; Rybak, 2007), with the exception that SOD activity is significantly increased (Gonzalez-Garcia et al., 2010).

In addition, ROS overload and depletion of antioxidant systems may allow cochlear lipid peroxidation as evidenced by the accumulation of malondialdehyde and toxic lipid peroxides, as well as aldehydes, such as 4-hydroxynonenal and peroxynitrite (Kopke et al., 1999; Lee et al., 2004a; Rybak, 2007).

### Inflammatory Reaction

Cisplatin increases the early immediate release and *de novo* synthesis of pro-inflammatory cytokines TNF-α, IL-1β, and IL-6 through the activation of ERK and NF-kB. However, antioxidants such as N-acetylcysteine (NAC) and GSH do not affect the secretion of pro-inflammatory cytokines, which suggests that inflammation is an upstream signal of ROS production (So et al., 2007). In another study, ROS generation through NADPH oxidase (NOX)3 triggered an inflammatory process by increasing the expression of COX-2, iNOS, and TNF-α in the cochlea through activation of signal transducer and activator of transcription-1 (STAT1) (Kaur et al., 2011). STAT6 signaling plays a pivotal role in cisplatin-mediated pro-inflammatory cytokine (IL-4 and IL-13) production and induction of ototoxicity (Kim et al., 2011).

### Apoptosis

ROS excess, lipid peroxidation, calcium influx, and inflammation can induce cellular apoptosis. Moreover, binding of cisplatin to DNA forms inter- and intra-strand cross-linking, which results in cytotoxicity (Casares et al., 2012). This pathological process induces death receptor (Devarajan et al., 2002; Jeong et al., 2007) and mitochondria-mediated (Garcia-Berrocal et al., 2007) apoptotic pathways. The pathways increase pro-apoptotic expression of Bax and decrease Bax-binding protein Bcl-2 family members, which change the permeability of mitochondrial membranes, releasing cytochrome c, activating caspase-9, and downstream caspase-3. The involvement of death receptor-dependent apoptosis is controversial in that caspase 8 is inactivated (Lee et al., 2004b; Garcia-Berrocal et al., 2007).

Further, cisplatin may induce ER stress in cochlear cells by activating caspase12 (an initiator caspase located on the ER membrane) and subsequently triggering apoptosis in a mitochondria-independent manner (Zong et al., 2017).

### Autophagy

Although autophagy is considered to be a contributor to cell survival through degradation of dysfunctional cytoplasmic components, it is also associated with cell death (McPhee et al., 2010). Autophagy differs from caspase-dependent apoptosis in that it is stimulated by oxidative and nitrosative stress (Filomeni et al., 2015). Both activation and inhibition of autophagy can reduce cisplatin-induced ototoxicity. For example, inhibition of ROS generation significantly prevents autophagy activation and apoptosis in response to cisplatin exposure (Yin et al., 2018). Further, enhancing autophagy can alleviate cisplatin-induced ototoxicity in rats (Liu et al., 2019) and zebrafish (Pang et al., 2018).

### Genetic Susceptibility to Cisplatin-Induced Ototoxicity

Candidate gene approaches support the impact of patients’ individual genotype on susceptibility of cisplatin-induced ototoxicity. These variants of genes are involved in DNA repair (XPC, eIF3a), detoxification of superoxide (SOD2) and transporters in the efflux of cisplatin (SLC31A1). Meanwhile, some genotypes (GSTs, SLC22A2) occur significantly more often among patients without ototoxicity, which show a protection against cisplatin-induced ototoxicity. These observations were made on small cohorts and independent replication is required. Additionally, high-throughput screens have been applied to search genomic biomarkers (TPMT, COMT, ABCC3, ACYP2) for cisplatin-induced hearing loss; however, independent cohorts for validation are necessary (Lanvers-Kaminsky and Ciarimboli, 2017; Drogemoller et al., 2019).

### Others

Nitrative stress could be another important factor in cisplatin-induced ototoxicity. Cisplatin treatment induces the nitration and degradation of cochlear protein LMO4 within the Organ of Corti, spiral ganglion, and stria vascularis (Jamesdaniel et al., 2012). Inhibition of nitration enables the transcription of STAT3-related anti-apoptotic genes in cisplatin-treated UB/OC1 cells (Rosati et al., 2019). Other nitrated proteins in the cochlear sensory epithelium have been discovered by proteomic analysis (Jamesdaniel et al., 2008). Furthermore, the activation of the iNOS pathway and the generation of nitric oxide is believed to facilitate ototoxicity (Watanabe et al., 2002; Li et al., 2006).

Parthanatos, a specific modality of cell death characterized by excessive activation of Poly (ADP-Ribose) Polymerase-1 (PARP1), is considered to be a cisplatin-induced ototoxic mechanism, which functions through PARP1-NAD^+^-SIRT1-NF-kB activation or a DNA damage-hearing loss pathway (Kim et al., 2016; Tropitzsch et al., 2019). Another programmed cell death pathway, necroptosis, has been demonstrated to contribute to cisplatin ototoxicity (Choi et al., 2019; Ruhl et al., 2019).

### Animal Models

Animal models are necessary for the study of cisplatin-induced ototoxicity. *In vitro* models such as auditory cell lines (HEI-OC1, UB-OC1, and VOT-E36) and explant cultures of the Organ of Corti are major tools for the evaluation of hair cell loss. In addition to zebrafish used for screening and optimizing potent therapeutic agents (Kros and Steyger, 2019), other *in vivo* animal models, particularly rodents (mice, rats, and guinea pigs), are utilized for histological analysis of auditory structures and physiological function.

### Intraperitoneal Injection (Single/Multi-Dose)

Cisplatin-induced hearing loss is associated not only with dosage, but also with the administration protocol. Whether used in single high dose or multiple low dose, one cycle or multiple cycles, consecutively or separately, different choices have different mortality rates and levels of hearing damage (Hughes et al., 2014; Harrison et al., 2016). The purpose of each is to establish a viable and stable ototoxicity model.

A single injected dose of cisplatin has been commonly used in that it is effective and practical at concentrations ranging from 10 to 30 mg/kg, as shown in **Table 1**. However, this approach is not similar to clinical chemotherapy in which cisplatin is administered as a single intravenous dose every 4 to 6 weeks with as many as six repetitions. Multi-dose administrations that mimic clinical regimens are essential. Three cycles of cisplatin with 10-day intervals has been established (Roy et al., 2013; Breglio et al., 2017) and optimized (Fernandez et al., 2019)to simulate clinical applications and minimize health risks and mortality, which also provide a better platform for development of novel therapeutic strategies.

**Table 1:** Summarized effects of otoprotective drugs on cisplatin-induced ototoxicity in various animal models and by differing delivery routes.

Drugs	Mechanisms	Animal model/cisplatin dose	Delivery route & dose	Outcomes	References
Apocynin	antioxidant	zebrafish, cisplatin 1mM	co-incubation, Apocynin solution, 125 and 250 mM, for 6 h	prevented hair cell loss at low concentrations	(Choi et al., 2013)
Lactate	antioxidant	guinea pigs, cisplatin I.P., 3 mg/kg/week x 8 weeks	intratympanic administration, Ringer’s solution, 0.5 h before cisplatin injection	reduced ABR threshold shift (averaged 17.0 dB), partial outer hair cell protection significant only at 2,000 Hz	(Nader et al., 2010)
Mitoquinone (MitoQ)	antioxidant	guinea pigs, cisplatin I.P., 10 mg/kg	subcutaneously (SQ) injection, 5 mg/kg/day x7 days and 1 h before cisplatin injection	reduced ABR threshold shifts (28-47 dB)	(Tate et al., 2017)
Paeoniflorin	antioxidant	mice, cisplatin I.P., 3 mg/kg/day x7 days	I.P., 30 mg/kg, for 2 h before daily cisplatin injection	increased SGN survival	(Yu et al., 2019)
Levosimendan	antioxidant	rats, cisplatin I.P., 15 mg/kg	I.P., 100 mg/kg/day x 5 days, starting 2 days before cisplatin injection	elevated signal-noise ratio (SNR) values, reduced cellular degeneration	(Gozeler et al., 2019)
Vitamin C	antioxidant	rats, cisplatin I.P., 16 mg/kg	intratympanic administration, 100 mg/ml, 0.5 h before cisplatin injection	decreased DPOAE amplitudes at 2.8, 4, 6, and 8 kHz frequencies	(Celebi et al., 2013)
Alpha-lipoic acid (ALA)	antioxidant	mice, cisplatin I.P., 20 mg/kg	I.P., 100 mg/kg of ALA for 2 days before/after cisplatin injection	almost completely protected hearing ability (5–10 dB change vs control)	(Kim et al., 2018a)
Ginkgolide B	antioxidant	rats, cisplatin I.P., 16 mg/kg	I.P., 10 mg/kg, immediately after cisplatin injection	reduced ABR threshold shift (about 20dB) at 16 and 32 kHz and inhibited vestibular dysfunction	(Ma et al., 2015)
Astaxanthin	antioxidant	rats, cisplatin I.P., 14 mg/kg	oral administration, 40 mg/kg daily through orogastric cannula before cisplatin injection	higher signal to noise ratios (SNRs) of DPOAE in high frequencies	(Kinal et al., 2019)
Flunarizine	anti-inflammation	mice, cisplatin I.P., 4 mg/kg/day x 4 days	oral administration, 143 μg/kg, 12 h before and at the same time as cisplatin delivery	attenuated pro-inflammatory cytokine secretion	(So et al., 2008)
R-phenylisopropyladenosine (R-PIA)	anti-inflammation	rats, cisplatin I.P., 11 mg/kg, using an infusion pump	intratympanic administration, 0.1 mM solution (50μl), 0.5 h before cisplatin injection	reduced ABR threshold shifts, especially at the highest frequency.	(Kaur et al., 2016)
Curcumin	antioxidant, anti-inflammatory	rat, cisplatin I.P., 16 mg/kg	I.P., 200mg/kg, 1 h before cisplatin administration and once daily for the following 3 days	decreased ABR thresholds by 20–25 dB at 6-32 kHz, increased DPOAE amplitude relative to cisplatin alone	(Fetoni et al., 2014)
Forskolin	antioxidant, anti-inflammation	mice, cisplatin I.P., 3 mg/kg/day x 7 days	I.P., 1mg/kg, one day ahead and at 2h before cisplatin injection	reduced ABR threshold shifts by 5–15 dB, especially at high frequency regions	(Guo et al., 2018)
Epigallocatechin-3-gallate (EGCG)	antioxidant, anti-inflammation	rats, cisplatin I.P., 11 mg/kg	oral administration, 100 mg/kg/day x 4 days, 1 day before cisplatin injection	reduced ABR threshold shifts (10-20dB) at 8, 16 and 32 kHz, attenuated loss of OHCs in the basal region, protected ribbon synapses and Na+/K+ ATPase α1 in SV/SL	(Borse et al., 2017)
Hydrogen (H2)	antioxidant, anti-inflammation, increase synaptophysin	guinea pigs, cisplatin I.P., 8 mg/kg	gaseous H2 inhalation (2% in air, 60 min), immediately after cisplatin injection	reduced ABR thresholds (-25dB) at 12.5, 20.0, and 30.0 kHz, attenuated OHC loss, protected IHC synapses	(Fransson et al., 2017)
N-acetylcysteine (NAC)	antioxidant, anti-apoptosis	rats, cisplatin I.P., 15mg/kg	I.P., 500 mg/kg/day x 3 days, 4 h after cisplatin on the first day	reduced ABR thresholds (-25dB) and increased DPOAE responses at all frequencies	(Somdas et al., 2018)
KR-22332	antioxidant, anti-apoptosis	rats, cisplatin I.P., 14 mg/kg	intratympanic administration, 2 mM, 0.5h before cisplatin injection	reduced ABR threshold shifts (-30dB) at 8kHz	(Shin et al., 2013)
Pifithrin‐α (PFT‐α)	anti-apoptosis	mice, cisplatin I.P., 16 mg/kg	I.P., 2.2 mg/kg, 0.5h before cisplatin injection on day 0 and daily for 5 days; I.T., 2 Mm (10μl), 0.5h before cisplatin injection on day 0 and daily for 2 days	reduced ABR threshold shifts from 4-32 kHz(20-25dB)	(Benkafadar et al., 2017)
Allicin	anti-apoptosis, anti-Parthanatos	mice, cisplatin I.P., 3 mg/kg/day x 7 days	I.P., 18.2 mg/kg, 1 day ahead and at 2 h before the injection of cisplatin	decreased ABR thresholds in most frequencies except 32 kHz, increased OHC and SGN survival, reduced apoptosis in SV	(Cai et al., 2019)
Minocycline	anti-apoptosis, anti-Parthanatos	guinea pigs, cisplatin I.P., 15 mg/kg	I.P., 45 mg/kg, 12h before cisplatin administration	reduced ABR threshold shift (10-20dB) in 16 kHz	(Lee et al., 2011)
Dunnione	anti-Parthanatos	mice, cisplatin I.P., 20 mg/kg,	oral administration, 20 mg/kg, 12h before cisplatin injection for the first dose, once a day for 4 consecutive days	attenuated ABR threshold shifts (20-25dB) at 4, 8, 16, and 32 kHz	(Kim et al., 2016)
Pirenzepine	anti-Parthanatos	cochlear explant cultures, cisplatin, 1.75µg/ml	co-incubation, 3-30μM	attenuated loss of sensory hair cells	(Tropitzsch et al., 2019)
Dexamethasone	maintain ion homeostasis and immune suppression	guinea pigs, cisplatin I.P., 8 mg/kg	intratympanic administration, 1 h before cisplatin injection and daily for 5 days	reduced ABR threshold (-40dB) in clicks at a range of 2-4kHz; preserved structure of HC&SC, SV&SL	(Shafik et al., 2013)
GMDTC	chelate platinum	mice, cisplatin I.P., 5 mg/kg, 2x weekly	I.P., 500 mg/kg, 2h after cisplatin and daily after	reduced ABR threshold shift at low frequencies	(Ge et al., 2019)
Kenpaullone	inhibit CDK2, antioxidant	mice and rats, cisplatin I.P., 30 mg/kg	intratympanic administration,310μM, 1h before cisplatin injection	reduced ABR threshold shifts (-10 dB) at 16 or 32 kHz	(Teitz et al., 2018)
AT7519 analogue 7 and AZD5438	inhibit CDK2	mice, cisplatin I.P., 10 mg/kg,	intratympanic administration, 50μM, 1h before cisplatin injection	AT7519-reduced ABR threshold shift at 16, 32 kHz (10 dB); AZD5438-reduced ABR threshold shift at 32 kHz (-14 dB), respectively	(Hazlitt et al., 2018)
Cimetidine	competitive OCT substrate	mice, cisplatin I.P., 15 mg/kg	I.P., 12.6 mg/kg, immediately before the cisplatin injection	reduced ABR threshold shifts at 16 and 32 kHz	(Ciarimboli et al., 2010)
ORC-13661	blocks MET channel	zebrafish and cochlear explant cultures, 5μM	co-incubation, ≥10μM, 48h	alleviated zebrafish lateral line and mammalian hair cell death	(Kitcher et al., 2019)
JWH015	activate endocannabinoid/CB2R system	rats, cisplatin I.P., 11 mg/kg	intratympanic administration, 2.5nM (50ul), 0.5h before cisplatin injection	reduced ABR threshold shifts (5-20dB) at 8,16,32 kHz, maintained the integrity of ribbon synapses and Na+/K+-ATPases in the SV	(Ghosh et al., 2018)
Capsaicin	activate endocannabinoid/CB2R system	rats, cisplatin I.P., 12 mg/kg	intratympanic administration, 0.1μM (50μl); oral administration, 10 or 20mg/kg, 24h before cisplatin injection	reduced ABR threshold shifts (-20dB) at 8, 16 and 32 kHz	(Bhatta et al., 2019)
Trichostatin A	regulate apoptosis, intracellular calcium homeostasis, neurotransmitter synthesis and release, and synaptic plasticity	cochlear explant cultures, cisplatin 150 mM	co-incubation, 200nM, pre incubation for 1h and co-incubation with cisplatin for 48h	reduced HC and SGN loss	(Wang et al., 2013)
Tauroursodeoxycholic acid (TUDCA)	promote endoplasmic reticulum (ER) proteostasis	rats, cisplatin I.P., 4.6 mg/kg/day x 3 days (day 1 3)	I.P., 100 mg/kg x 5 days (day 0-5)	reduced ABR threshold shift (18–28 dB) at 8–32 kHz	(Kim et al., 2018b)
Fenofibrate	maintain functional peroxisomes and mitochondria, antioxidant	mice, cisplatin I.P., 4 mg/kg/day x 4 days	I.P., 50mg/kg, 12h before cisplatin for first dose, immediately after cisplatin treatment for second dose and repeated for four consecutive days	reduced ABR threshold shifts (-10dB) at 4–32 kHz	(Kim et al., 2018b)

Significant ototoxic differences have been found for different rodent species. For example, guinea pigs exhibit an increased sensitivity to cisplatin compared with mice (Poirrier et al., 2010). Among different mouse strains (CBA/CaJ, C57BL/6J, BALB/cJ mouse), the BALB/cJ strain had the greatest threshold shift after cisplatin injection and the lowest mortality (DeBacker et al., 2020). It suggests the need for careful selection of animal species.

### Local Exposure

The large inter-animal variability observed with systemic drug administration can be overcome by direct administration of cisplatin into the cochlea *via* an osmotic minipump system (Wolters et al., 2003). Although this intracochlear application is not clinically practical, which is the major drawback of this protocol, the animal model is ideally suitable for studies on otoprotective interventions. On the other hand, the delivery site of cisplatin at basal turn can be an interference factor of the intrinsic base-to-apex gradient in hair cell loss (O’Leary et al., 2001). Additionally, the trans-tympanic route lowers morbidity and dose-dependent cochlear or vestibular toxicity (Callejo et al., 2017).

## Strategies to combat Cisplatin-Induced Ototoxicity

In recent decades, preclinical pharmacological strategies have assessed means by which to reduce the ototoxic effects of cisplatin. However, none have been approved by the FDA. These assessments have been based on the underlying mechanisms identified above. The following describes promising small molecule compounds, novel delivery systems, and routes of delivery in a variety of experimental animal models.

### Small Molecule Compounds

Various studies have assessed the potential protective effects of compounds. These include blockade of cisplatin entry into the cochlear fluid or hair cells. Further, antioxidants are among the most extensively studied agents due to the importance of ROS in ototoxicity. However, a concern exists that the chemotherapeutic efficacy of cisplatin may be reduced (Block et al., 2009). Reductions in inflammatory cytokine levels have been another target for drug development by which to protect from cisplatin-induced hearing loss. Finally, strategies to reduce apoptosis or other forms of cell death have shown great promise. **Table 1** identifies otoprotective candidates evaluated in the last 10 year, as well as operative pathways by which cochlear cell cytotoxicity is induced.

### Drug Delivery Routes

One of the most important choices for a protective intervention is the route of drug delivery. The amount and distribution of drug depends both on the substance applied and on the application protocol. Pharmacological therapy to the inner ear can be divided into two forms: systemic or local administration. Both have pros and cons.

#### Systemic Administration

Systemic administration is a practical and less invasive method for delivery by oral, intravenous, intraperitoneal (I.P.), or subcutaneous routes. As shown in **Table 1**, these methods of administration are preferable and have protective effects against cisplatin-induced ototoxicity. Among these compounds, many have been applied systemically before the injection of cisplatin for a single dose (Paeoniflorin, Levosimendan) or multi doses (Curcumin, Dunnione), while some are used after cisplatin injection (Ginkgolide B, N-acetylcysteine). For low-frequency hearing loss, associated with the cochlear apex, systemic administration provides for relatively uniform drug distribution along the cochlea. For high-frequency hearing loss local administration is appropriate (Wang et al., 2018b).

However, there are two challenges. The first is to overcome biological barriers that restrict access to the inner ear. These are the blood-perilymph, blood-endolymph, perilymph-endolymph, and middle-inner ear barriers (Zou et al., 2016). The second is off target side effects of systemic administration and the possibility of drug clearance prior to target site access. Unwanted side effects include hematological changes (Freyer et al., 2017) and decreased antitumor efficacy (Lanvers-Kaminsky et al., 2017).

#### Local Administration

The alternative to systemic administration is local drug delivery, which mainly includes intratympanic and intracochlear/intralabyrinthine delivery (Anderson et al., 2019). Advantages of local delivery include passage through the blood-labyrinth barrier, acquisition of higher drug concentration in the cochlea, and avoidance of “first-pass” metabolism (Plontke et al., 2014). Further, the development of 3D computer simulated delivery and sampling procedures have provided a valuable tool to interpret the amount and distribution of drug within the ear (Salt and Plontke, 2018).

##### Intratympanic (I.T.) Delivery

Intratympanic injections of therapeutics (Kenpaullone, JWH015, Capsaicin, etc.) are the most commonly-used local delivery and many of which have been proven effective in hearing protection. Drugs are commonly applied to the round window (RW) niche where it contacts both the round window membrane (RWM) and the stapes footplate. If not cleared through the eustachian tube, higher drug concentrations are found in the scala vestibuli rather than the scala tympani. A decreasing basal-apical drug concentration gradient along the cochlea may limit the therapeutic effect of the drug (Plontke et al., 2008). Caution must be exercised in that vestibulotoxicity can develop with prolonged drug retention (Salt et al., 2016) and cochleotoxicity with higher dosage (Okuda et al., 2004) and conductive hearing loss. Moreover, multi-cycle intratumor administration may be required, which can damage tissue and bone and is expensive.

The RW is also a site for chronic drug delivery by a combination of micropump and catheter over a long time course, which provides predictable duration of delivery and consistent drug concentration (Sale et al., 2017). Potential adverse local effects are middle ear granulation (Plontke et al., 2006) and inflammation of the RWM. Furthermore, obstruction (pseudomembrane, fibrous, or fat plug) of the RWM (Alzamil and Linthicum, 2000) is another obstacle to local delivery.

##### Intracochlear/Intralabyrinthine Delivery

Intracochlear administration has the advantage of passage through physiological barriers. Compared with I.T. delivery, intracochlear drug delivery systems have the advantages of improved dosing control and reduced drug concentration gradients (Pierstorff et al., 2019). Varied approaches have been developed to improve efficacy including the use of sealing materials to reduce leakage while injecting through the RWM (Plontke et al., 2016) and the application of osmotic minipumps to achieve chronic delivery (Wang et al., 2004; Wolters et al., 2004). Gene therapy and cell transplantation by intracochlear delivery hold future promise for treatment (Salt and Plontke, 2018).

#### An Optimal Delivery Pattern

To summarize, the most suitable protocol for treating cisplatin-induced ototoxicity is intratympanic injection to be delivered 1h before each cisplatin injection, due to superior perilymph concentrations within 1 h of administration (Chandrasekhar et al., 2000; Hargunani et al., 2006). I.T. delivery has no effect on anticancer effect of the cisplatin by eliminating systemic absorption. While the major obstacle is the trama of middle ear and intratympanic pain, which can be relieved by advanced technique of endoscope and analgesia.

### Delivery Systems

Many strategies for both systemic and local delivery to the inner ear have been developed to improve local effectiveness and to reduce systemic side effects. Innovations in drug delivery systems, including nanoparticles, hydrogels, and environmental stimuli systems have been applied to the inner ear.

#### Nanoparticles

Nanoparticles (NPs), characterized by a diameter of < 1μm, provide many means by which to deliver drugs to the inner ear, including liposomes, polymer nanoparticles, lipid emulsions, nanocapsules, and solid lipid NPs (Mader et al., 2018). After intratympanic administration, drug can reach the cochlea through the RWM by diffusion of the drug-loaded NPs or by diffusion of the free drug released from NPs (Mader et al., 2018). Initially, NPs with sizes < 200 nm or with high lipid solubility were thought to penetrate the RWM (Bowe and Jacob, 2010). However, particle size, surface chemistry, and cell-penetrating peptides (CPPs) all have impact on cochlear drug delivery* in vivo* (Cai et al., 2017). Specifically, NPs with sizes of 150–300nm, positive surface charge and some particular CCPs (low molecular weight protamine, LMWP) provide an enhancement in cochlear entry (Cai et al., 2017).

Various materials have been used for NP construction, including lipids, inorganic materials (such as gold, carbon or iron), proteins, and polymeric systems (Morachis et al., 2012). Each have a different capacity to reduce cisplatin-induced ototoxicity. For example, Martin-Saldana (Martin-Saldana et al., 2016) designed an NP that not only encapsulated a functional drug (methylprednisolone) but also was active in itself as the NP was constructed of a methacrylic derivatives of α-tocopherol (vitamin E). Biodegradable and biocompatible solid lipid nanoparticles (SLNs) are able to increase glucocorticoid dose to the inner ear, improving protection (Cervantes et al., 2019). Hydrophobic agents with short half-lives, such as dexamethasone and α-tocopheryl succinate, have been incorporated into NPs with adequate concentrations that reach the inner ear (Martin-Saldana et al., 2017). Moreover, modification to NP surfaces can improve desirable attributes. For example, A666 peptide-conjugated NPs specifically target prestin in OHCs with anticipated otoprotective activity (Wang et al., 2018a).

#### Hydrogel

Solutions administered intratympanically tend to be absorbed through the eustachian tube and cleared through the middle ear mucosa, which provides limited temporal exposure to the inner ear. This obstacle is overcome by the use of hydrogel. OTO-104(poloxamer hydrogel containing dexamethasone) provides a sustained-exposure to dexamethasone and alleviates cisplatin-induced ototoxicity (Fernandez et al., 2016). A Diltiazem (calcium-channel blocker)-loaded chitosan-glycerophosphate (CGP) hydrogel has been used as a vehicle to provide controlled and sustained delivery to the inner ear (Naples et al., 2020). In addition, hydrogel itself can be cross-linked with functional reagents, such as genipin or STS, with potential therapeutic effect (Videhult Pierre et al., 2019; Yuksel Aslier et al., 2019). For example, silk fibroin and homogenously deacetylated chitosan formulations undergo spontaneous transformation from an aqueous phase to gel and provide rapid transport to the inner ear and prolonged release through the RWM (Chen et al., 2019; Videhult Pierre et al., 2019). In spite of these attractive properties, there is a concern for subsequent conductive hearing loss due to hydrogel attachment to the RWM. A clinical trial has demonstrated I.T. administration of STS-hyaluronate gel to be feasible and safe with mild adverse effects, although the protection outcomes were not statistically nor clinically significant (Rolland et al., 2019).

#### Environmental Stimuli Systems

The major constraint on effective nanoparticle delivery is their poor cellular internalization. Given this, environmental stimuli systems may increase their effective delivery. The stimuli systems can be divided into endogenous (redox, pH, enzyme) and exogenous (light, heat, magnetic field, and ultrasound) types (Morachis et al., 2012). The former ones occur inherently and are beneficial for clinical application. A designed pH-sensitive polymeric nanoparticle system, triggered by an acid environment due to increased ROS and inflammation, can release encapsulated dexamethasone and ameliorate hearing loss by intratympanic administration (Martin-Saldana et al., 2018). Endogenous stimuli are favored by systemic administration and only activated for regulated release at specific sites (Movahedi et al., 2015). In a magnetic field, nano-constructs consisting of superparamagnetic iron oxide nanoparticles (SPIONs) entrapped within glutathione micelles can be used to sequester extracellular cisplatin before it enters a cell (Martin-Saldana et al., 2017). NPs with encapsulated prednisolone can be delivered magnetically to the cochlea with substantial reduction in hearing loss (Ramaswamy et al., 2017). *In vitro*, a photosensitive substance, 4-azidosalicylate activated by UV light, has been used to disable prestin in in isolated OHCs, resulting in permanent electromotility inhibition (Fisher et al., 2012). Although optogenetic applications have not been used to treat cisplatin-induced ototoxicity, possibilities have been described (DiGuiseppi and Zuo, 2019).

#### Others

Drug delivery carriers are also suitable for use with cisplatin, including NPs, liposomes, micelles (Baba et al., 2012), and nanocapsules (Boulikas, 2009; Vhora et al., 2014). These can selectively and effectively accumulate in solid tumors, enhancing anticancer potential and reducing toxicity.

## Clinical Trials

Although an extensive number of preclinical studies have explored protective interventions to reduce cisplatin-induced ototoxicity, there are no generally established clinical guidelines. The challenges for translation of preclinical to clinical trials are: (1) differences in morphology and physiology between the human cochlea and that of experimental animals (Laurell, 2019); (2) identification of ototoxic susceptible patients in that international standards among classification systems do not exist (Knight et al., 2017).

Several well-studied laboratory protective approaches have been extended to the clinic. Two randomized controlled trials (RCTs) have verified the otoprotective effect of intratympanic dexamethasone and N-acetylcysteine, which included attenuation of hearing loss and alleviation of OHC dysfunction (Marshak et al., 2014; Sarafraz et al., 2018). Another antioxidant, amifostine, significantly reduced cisplatin-induced serious hearing loss in patients with average-risk medulloblastoma (Fouladi et al., 2008; Gurney et al., 2014). Some trials have had contradictory results (Yoo et al., 2014; van As et al., 2016) or protective failure, (Fox et al., 2018), attributed to the difference in drug dosage and timing for administration prior to cisplatin treatment, which might decrease the potential maximal protective effect of the same drug. Therefore, a larger scale research, employing various concentrations to be delivered in precise timing, is required.

Notably, sodium thiosulfate is a promising agent. In a recent multicenter, randomized, phase 3 clinical trial (NCT00652132) (Brock et al., 2018), patients with hepatoblastoma who received STS 6 h after the cisplatin infusion had a 48% lower incidence of hearing loss than the cisplatin-alone group. And there was no significant difference in 3-year rates of event-free survival (82% vs 79%) or overall survival (98% vs 92%) between the two groups. Similarly, in another clinical trial (NCT00716976) (Freyer et al., 2017), STS treatment reduced the cumulative incidence of cisplatin-induced hearing loss nearly by half (28.6% vs 56.4%), without jeopardizing overall or event-free survival in participants with localized disease. These results suggest a new era with encouraging possibilities in cases of inevitable cisplatin chemotherapy due to its efficacy and safety.

## Opportunities and Challenges

To reduce cisplatin-induced ototoxicity, there are challenges, including (1) an incomplete understanding of the underlying mechanisms of ototoxicity, (2) selection of an optimal strategy (when to use and by which route) among many alternatives, (3) translation to clinical application.

There is a need to identify the pathogenic basis for cisplatin-induced ototoxicity. This identification will guide future advances in effective otoprotective agents. Systemic administration of sodium thiosulfate may have a promising future and ‘cocktail treatments’ (multi-targeted drug combination) may potentially activate multiple molecular pathways. Moreover, identification of pharmacogenomic markers may reduce cisplatin-induced ototoxicity by identifying patients at greatest risk, or who require closer audiologic monitoring, or may benefit from another platinum derivative, or require a reduced drug dosage. In all cases, interference with antitumor efficacy must be considered. Importantly, an internationally approved strategy needs to be implemented for clinical practice.

## Author Contributions

JG, YC, and WK wrote the draft. DY, XW, and HW amended the draft.

## Funding

This work was supported by the National Natural Science Foundation of China (No. 81970874, No. 81700899), the Shanghai Municipal Science and Technology Commission (No.19ZR1429400), the Shanghai Municipal Education Commission – Two-hundred Talent (No. 20171919), and the Interdisciplinary research of 9th People’s Hospital affiliated to Shanghai Jiao Tong university School of Medicine (No. JYJC201810).

## Conflict of Interest

The authors declare that the research was conducted in the absence of any commercial or financial relationships that could be construed as a potential conflict of interest.

## Abbreviations

ABR, auditory brainstem response; CCPs, cell-penetrating peptides; DPOAE, distortion product otoacoustic emission; EP, endocochlear potential; HC/SC, hair cell/supporting cell; I.P., intraperitoneal; I.T., intratympanic; MET, mechanoelectrical transducer; NOX3, NADPH oxidase 3; NPs, nanoparticles; RCT, randomized controlled trial; ROS, reactive oxygen species; RWM, round window membrane; SR/ER, sarcoplasmic/endoplasmic reticulum; SV/SL, stria vascularis/spiral ligament.
